# Head and neck cutaneous basal cell carcinoma: a retrospective analysis of tumour features, surgical margins and recurrences

**DOI:** 10.1007/s00405-025-09216-z

**Published:** 2025-02-20

**Authors:** Pasquale Di Maio, Marco Giudice, Antonio Cavallero, Claudio Carnevale, Guillermo Til-Pérez, Pedro Luis Sarría-Echegaray, Chiara Copelli, Guglielmo Ramieri, Oreste Iocca

**Affiliations:** 1 Doctoral Degree in Translational Research in Public Health and High Prevalence Diseases, UIB - Universitat de les Illes Balears, Palma de Mallorca, Spain; 2 Department of Otolaryngology-Head and Neck Surgery, Giuseppe Fornaroli Hospital, ASST Ovest Milanese, Via al Donatore di Sangue, 50, Magenta, Milan, 20013 Italy; 3 Department of Otolaryngology-Head and Neck Surgery, Giovanni Borea Civil Hospital, Sanremo, Italy; 4 Servicio de otorrinolaringologia y cirugía de cabeza y cuello, Clínica Rotger, Palma de Mallorca, Spain; 5 Facultad de Medicina, Universidad de las Islas Baleares, Palma de Mallorca, Spain; 6 Division of Maxillofacial Surgery, Department of Interdisciplinary Medicine, University of Bari, Bari, Italy; 7 Division of Maxillofacial Surgery, City of Health and Science of Turin Hospital, University of Turin, Turin, Italy

**Keywords:** Non-melanoma skin cancer, Basal cell carcinoma, Head and neck cancer, Surgical margins, Recurrence

## Abstract

**Purpose:**

The aim of this study was to analyse the factors affecting the status of surgical margins and recurrence of basal cell carcinoma (BCC) of the head and neck. A secondary aim was to provide detailed demographic, clinical and topographic data to understand the biological behaviour of this skin cancer in head and neck area.

**Methods:**

A retrospective analysis was conducted analysing all primary head and neck BCCs treated from July 2014 to October 2021. Chi-square and logistic regression were used to assess the presence of statistically significant associations.

**Results:**

The study cohort included 307 patients who underwent resection of 377 BCCs. The mean age of the patients was 76.86 years. There were 251 (67%) clean surgical margins, 80 (21%) positive and 46 (12%) closed. Recurrences were observed in 11 (5%) out of 218 BCCs of patients with a minimum follow-up of 24 months. The median follow-up time was 35 months. Positive margin status was significantly associated with BCC of the nose, while clean margin was correlated with neck localization (p<0.05). Clean margin status was linked with direct closure (p<0.05), while positive and closed margins were significantly associated to local flaps (p<0.05). Positive margin status was significantly related to depth of invasion below the dermis (p<0.05).

**Conclusion:**

The location, depth of invasion and method of reconstruction of head and neck BCC influence the completeness of surgical resection. Considering the low recurrence rate, clinical observation is an acceptable management option in patients with compromised margins, especially in elderly and frail patient populations.

**Supplementary Information:**

The online version contains supplementary material available at 10.1007/s00405-025-09216-z.

## Introduction

Cutaneous basal cell carcinoma (cBCC) is the most common malignant neoplasm diagnosed worldwide, with a steadily increasing growth rate, due to more frequent exposure to natural and artificial ultraviolet (UV) radiation, change in clothing style and raised life expectancy. Clinically, cBCC presents as a slow-growing and locally invasive lesion, it rarely metastasizes and has an extremely low mortality rate. However, although the tumor prognosis is generally favorable, its high incidence rate carries a significant socioeconomic burden, often underestimated as cBCCs are frequently excluded from cancer registries [1–2].

This non-melanoma skin cancer (NMSC) originates from the basal cells of the epidermis and has exposure to UV radiation as its main risk factor. Consequently, it typically occurs in areas of the body that are chronically exposed to the sun. For this reason, the head and neck is the site where cBCC occurs most often (~ 80%) [1, 3]. Surgical removal is often the curative treatment of cBCC, especially if diagnosed in the early stages. In the head and neck area, where function and esthetics are considerable concerns, surgery turns out to be a compromise between safe excision margins and satisfactory cosmetical results [4]. For this reason, the facial region represents the site in which the cBCCs are most frequently removed incompletely [5–6], with a frequency of compromised margins ranging from 9% to 37,2% [7].

This retrospective study describes a series of head and neck BCCs treated in a tertiary care hospital with the aim to analyze factors influencing surgical margin status and tumor recurrence. Also, demographic, clinical, and topographic details are provided.

## Methods

The Department of Otolaryngology and Head and Neck Surgery of Giovanni Borea Civil Hospital of Sanremo, based in north-west Italy, provides a non-melanoma skin cancer (NMSC) service for cutaneous lesions of the head and neck. A retrospective clinical and pathological analysis of the institutional database was performed including all patients treated between July 2014 and October 2021 for BCC of the head and neck region. To exclude cases of suspected melanoma, all patients underwent a dermatological consultation before excision. All lesions were treated with once-off excision under local anaesthetic (1% lidocaine), with no previous biopsy, marking them before removal to include a surgical clearance margin of at least 2 mm. This approach is supported by the results of a recent network meta-analysis, which identified primary excision as the preferred initial management of NMSCs [8]. Patients with incomplete data, non-primary BCCs and those undergoing adjuvant treatment were excluded. Only patients with a minimum follow-up of 24 months were included for the analysis of recurrences.

Recorded data were age, sex, anatomical distribution within head and neck, side, tumour size, histological type, deep invasion, incomplete margins (lateral, deep or both), surgeon’s experience, excision of more lesions in the same session, surgical wound closure method, positive margin management, follow up, recurrences and their management. BCCs were classified histologically into six subtypes: superficial, nodular, micronodular, infiltrative, morpheaform, and metatypical (basosquamous). When more than one subtype was present in the same lesion, the BCC was classified as mixed. Surgical experience was evaluated based on the number of BCC excision procedures performed by the physician throughout his/her career and classified into 3 groups: 1) < 100 procedures, 2) ≥ 101 < 300 procedures and 3) ≥ 301 procedures. The surgical procedures were performed by 7 consultants, two belonging to the first group, three to the second and two to the third.

The case cohort collected was divided into 3 classes: “clean”, “positive” and “close” margins. A pathological margin of less than one millimetre was considered a close skin margin [9–10]. The closed margins were always managed with clinical observation [11], while the positive margins with observation or re-excision according to age, comorbidities and patient choice after adequate counselling.

Chi-square tests were performed for categorical variables and analysis of adjusted residuals was performed to better interpret statistically significant results. In detail, it has been evaluated if the margins status after excision was significantly associated to the experience of the surgeon, multiple excisions surgery, primary anatomical site, side of the tumour, depth of invasion and closure method. Also, it was assessed if tumour recurrence was significantly associated to margin status, primary site, depth of invasion and method of closure. Furthermore, it was analysed if the localization of the positive margin (lateral, deep, or both) was associated with recurrence. Logistic regression was used to estimate correlation of tumour diameter with the outcome positive margin ‘lateral’, ‘deep’, or ‘both’. Also, the same was done to evaluate a correlation of tumour diameter with the outcomes margin ‘close’, ‘positive’ or ‘clean’. Kaplan-Meier curve for patients with a minimum follow-up of 24 months was constructed to study the recurrences. All tests were two sided. Statistical significance was set as *p* < 0.05. Analyses were performed with SPSS 21.0 (SPSS inc).

## Results

The study population included 307 Caucasian patients who underwent resection of 377 primary head and neck BCCs, most of whom were referred by the dermatology department. Male patients were 193 (63%) and females 114 (37%), with a mean age of 76,86 years (SD 10,03, range 41–97). Regarding the age distribution of patients at the time of BCCs removal: 7 (2%) of patients were in the age group 40–49 years, 19 (5%) in 50–59 year., 45 (12%) in 60–69 year., 142 (38%) in 70–79 year., 141 (37%) in 80–89 year., and 23 (6%) were 90 year. of age or older. In the study period 251 patients (82%) had only one BCC removed, 47 (15%) two, 6 (2%) three, 2 (0.5%) four and 1 (0.5%) five BCCs.

Regarding anatomical location, the most frequent subsite was the nose where BCC occurred in 132 (35%). Ear and preauricular area were affected in 105 (29%) cases, cheek and zygomatic area in 51 (13%), scalp in 47 (12%), eye area in 21 (6%), the perioral area in 13 (3%) and the neck in 8 (2%). One hundred and fifty-three (41%) were located on the right side of the face, 150 (40%) on the left side, 57 (15%) were on the midline, while 17 (4%) could not be defined the side. The mean size of the tumour specimens, defined as maximum diameter, was 9.3 mm (SD 6.24, range 1–45). Complete description of demographic, anatomical and histological characteristics is presented in Table 1.

**Table 1:** Demographic, anatomical and histological notes

Characteristics	No. of patients (*N* = 307)
Mean age, y (SD)	76,86 (10,03)
Sex (%)	
Male	193 (63)
Female	114 (37)
**Characteristics**	**No. of BCCs (** *N* ** = 377)**
Location (%)	
Nose	132 (35)
Ear/Preauricular area	105 (29)
Cheek/Zygomatic area	51 (13)
Scalp	47 (12)
*Forehead* *Temporal* *Remaining scalp’s areas*	20 19 8
Eye area	21 (6)
Perioral area	13 (3)
*Upper lip* *Lower lip* *Chin*	8 1 4
Neck	8 (2)
Side (%)	
Right	143 (41)
Left	150 (40)
Midline,	57 (15)
NA	17 (4)
Tumour diameter	
mm (SD)	9.3 (6.24)
Histology (%)	
Nodular	147 (39)
Micronodular	12 (3)
Superficial	9 (2)
Infiltrative	25 (7)
Sclerosing	39 (10)
Morpheaform	28 (8)
Metatypical	14 (4)
Mixed histology	23 (6)
NOS/Indeterminate	80 (21)
Perineural invasion (%)	
Yes	2 (0,5)
No	375 (99,5)

Abbreviation: NA, not available; NOS, not otherwise specified

Histological evaluation of the surgical margins found that 251 (67%) were clean, 80 (21%) positive and 46 (12%) close. For the positive margins the most affected were the lateral ones in 52 (65%), followed by deep in 18 (22%), while in 10 (13%) cases both (lateral and deep) the margins were positive. The same occurred for the close margins, with an involvement of the lateral ones in 30 (65%), of the deep ones in 13 (28%) and of both in 3 (7%). Compromised margins were more frequent on the nose. In detail, the nose had positive margins in 39 (49%), the auricular area in 16 (20%), the scalp in 11 (14%), the cheek and zygomatic area in 9 (11%), the ocular in 3 (4%) and perioral in 2 (2%). The close margins were found in 20 (44%) on the nose, in 16 (35%) on the ear area, in 4 (9%) on the scalp, in 2 (4%) on the cheek and cheek area, in 2 (4%) on the ocular area and in 2 (4%) on the perioral area. There were no cases of compromised margins on the neck.

Wound management was implemented primarily by direct closure in 244 (65%) patients, followed by local flaps in 122 (32%) and full-thickness skin grafts in 11 (3%). The 80 positive margins were handled in most cases with close clinical observation, in 3 (4%) cases with surgical re-excision. In these three cases, the histological examination of the re-excision specimens resulted negative for residual BCC. Table 2 summarizes the aforementioned results.

**Table 2:** Margin features and wound management

Margin status (%)	No. of tumours (*N* = 377)
Clean margins	251 (67)
Positive margins	80 (21)
Close margins	46 (12)
**Positive margin type (%)**	
Lateral	52 (65)
Deep	18 (22)
Both	10 (13)
**Close margins type (%)**	
Lateral	30 (65)
Deep	13 (28)
Both	3 (7)
**Positive margins site (%)**	
Nose	39 (49)
Auricular area	16 (20)
Scalp	11 (14)
Cheek/Zygomatic	9 (11)
Ocular area	3 (4)
Perioral area	2 (2)
**Close margins site (%)**	
Nose	20 (44)
Auricular area	16 (35)
Scalp	4 (9)
Cheek/Zygomatic	2 (4)
Ocular area	2 (4)
Perioral area	2 (4)
**Wound closure (%)**	
Primary closure	244 (65)
Local flaps	122 (32)
Skin grafts	11 (3)

Tumour recurrences were evaluated only in patients with a minimum of 24 months of follow-up. In this subgroup of patients, 11(5%) recurrences were identified out of 218 BCCs. Of these eleven in 6 cases the primary excision margins were clean, in 1 close and in 4 positives. Therefore, the recurrence rate was 4% (6/141) in the clean margin group, 8% (4/48) in the positive margins and 3.5% (1/29) in the closed margins. Mean time to recurrence was 23.7 months (SD 9.44, range 4–33, 95% confidence interval [CI] 17.3–30 months). Median time to recurrence was 24 months (interquartile range 14 months). Within the 1st year of follow-up 18% (2/11) of recurrences occurred, 36% (4/11) during the 2nd year and 46% (5/11) during the 3rd year. All clinical recurrences were treated with additional surgical resection and tested positive for BCC. Out of 11 recurrences, only one case 9%of second recurrence was observed after 14 months from the first, also treated with surgical excision. Median total follow up time was 35 months (interquartile range 18 months). No cases of distant or regional metastases were recorded during follow up. Characteristics of recurrent tumours are detailed in Table 3.

**Table 3:** Clinical features of recurrent BCC

	Gender	Age	Location	Histological subtype	Recurrence time
1	M	82	Ear/pre-auricular area	Nodular	23
2	M	87	Nose	Nodular	33
3	F	76	Nose	Nodular	33
4	M	81	Nose	Nodular	18
5	F	75	Nose	Morpheaform	33
6	F	79	Nose	Nodular	24
7	M	88	Ear/pre-auricular area	Sclerosing	4
8	M	85	Nose	Nodular	28
9	F	66	Nose	Nodular	22
10	M	87	Ear/pre-auricular area	Morpheaform	31
11	M	79	Cheek/Zygomatic	Morpheaform	12

Margin status was not associated significantly to surgical experience (p 0.655), excision of multiple lesions in the same surgical session (p 0.439) and side of the tumours (p 0.886). The method of wound closure was connected with margin status (p 0.004). In detail, clean margin status was associated with direct closure (adjusted residual 3,3), while the use of local flaps with positive and close margins (adjusted residual 2,1 and 2,0– respectively). Also, margin status was related significantly with primary tumour site (p 0.046). Particularly, positive margin status was significantly associated with BCC localized on the nose (adjusted residual 2,9), while clean margin status with neck localization (adjusted residual 2.1). Furthermore, positive margin status was significantly linked to depth of invasion below the dermis (p 0.045; adjusted residual 2.5). Table 4 summarizes the influence of the analysed variables on the margin status. Logistic regression analysis showed that tumour diameter was not correlated with the outcome of the margin being lateral, deep or both (*p* > 0,05), or with the outcome close, positive or clean (*p* > 0.05).

**Table 4:** BCC features and factors affecting margin status

Status of the margins	Clean *n* = 251 (67%)	Close *n* = 46 (12%)	Positive *n* = 80 (21%)	Total	*p* value
Side of tumor Right Left Midline Missing	96 (63%) 101 (67%) 39 (68%) 15 (88%)	22 (14%) 17 (11%) 7 (12%) 0 (0%)	35 (23%) 32 (22%) 11 (20%) 2 (12%)	153 150 57 17	0.886
Site Nose Ear/preauricular Cheek/zygoma Scalp Eye area Perioral area Neck	73 (55%) 73 (70%) 40 (78%) 32 (68%) 16 (76%) 9 (70%) 8 (100%) *	20 (15%) 16 (15%) 2 (4%) 4 (8%) 2 (10%) 2 (15%) 0 (0%)	39 (30%) * 16 (15%) 9 (18%) 11 (24%) 3 (14%) 2 (15%) 0 (0%)	132 105 51 47 21 13 8	0.046 *
**Reconstruction** Primary closure Local flap Skin graft	177 (73%) * 68 (56%) 6 (55%)	23 (9%) 21 (17%) * 2 (18%)	44 (18%) 33 (27%) * 3 (27%)	244 122 11	0.004 *
**Depth of invasion** Epidermis/dermis Below dermis	235 (68%) 16 (52%)	43 (12%) 3 (10%)	68 (20%) 12 (38%) *	346 31	0.045 *
**Surgical experience** < 100 procedures ≥ 101 < 300 procedures ≥ 301 procedures	44 (68%) 120 (69%) 87 (63%)	8 (12%) 17 (10%) 21 (15%)	13 (20%) 36 (21%) 31 (22%)	65 173 139	0.655
**Multiple excisions** Yes No	55 (75%) 196 (65%)	6 (8%) 40 (13%)	12 (17%) 68 (22%)	73 304	0.439

*statistically significant value

Regarding recurrences, analysis on patients with at least 24 months of follow-up showed that margin status was not significantly associated with a recurrence (p 0.491), although a tendency toward a correlation of recurrence and positive margins was observed (adjusted residual 2.1). The localization of the positive margin (lateral, deep, or both) was not associated with recurrence (p 0.863). Primary tumour site (p 0.160) and depth of invasion (p 0.788) were not associated with recurrence; although a trend was identified with the correlation of recurrence with nose localization (adjusted residual 2.2). Method of closure of the surgical wound was not significantly associated with the recurrence (p 0.212). The analysis of factors affecting tumour recurrence is recapped in Table 5. Kaplan_Meier curve for recurrence survival is depicted in supplementary Fig. 1s, with a mean recurrence free survival of 84.9 months (95% CI 82.6–87.3).

**Table 5:** BCC features and factors affecting recurrences in patients with 24 months of minimum follow up

Recurrence	Yes *n* = 11 (5%)	No *n* = 207 (95%)	Total 218	*p* value
Site Nose Ear/preauricular Cheek/zygoma Scalp Eye area Perioral area Neck	7 (8%) 3 (5%) 1 (4%) 0 (0%) 0 (0%) 0 (0%) 0 (0%)	74 (92%) 59 (95%) 23 (96%) 29 (100%) 11 (100%) 8 (100%) 3 (100%)	81 62 24 29 11 8 3	0,551
Margins Clean Close Positive	6 (4%) 1 (3%) 4 (8%)	135 (96%) 29 (97%) 44 (92%)	141 29 48	0.491
**Location of the positive margin** Lateral Deep Both	3 (8%) 1 (12%) 0 (0%)	35 (92%) 7 (98%) 3 (100%)	38 8 3	0.790
**Reconstruction** Primary closure Local flap Skin graft	7 (5%) 3 (4%) 1 (20%)	135 (95%) 68 (96%) 4 (80%)	142 71 5	0.296
**Depth of invasion** Epidermis/dermis Below dermis	10 (5%) 1 (5%)	188 (95%) 19 (95%)	198 20	0.992

## Discussion

The incidence of NMSC has significantly increased around the world in recent decades, mainly due to an aging population, as well as social changes and medical conditions; among the latter play a predominant role immunosuppressed states that make patients more prone to develop this type of skin cancer. In addition to constituting over 80% of NMSCs, BCC is the most diagnosed cancer in Caucasian populations, posing a major issue and financial burden for healthcare systems due to its constant raise. The cervicofacial district is the body area most affected by the BCC, this means that head and neck surgeons are often involved in its therapeutic management, surgery being the main treatment procedure [1, 12–13].

Our study cohort consisted mainly of male patients (63%), likely due to factors related to occupational exposure to sunlight [13]. In fact, even if it was not possible to investigate in detail on each patient’s work related data, many patients were fishermen or flower growers, both activities among the main ones carried out in this geographical area of Liguria. Head and neck BCC was most prevalent in the 70–79 (38%) and 80–89 (37%) age groups with a mean age at presentation of 76.86 years. These data on the prevalence of age in the seventh decade is in line with other studies conducted in our country [12, 14], but differ from those of other Mediterranean countries which reported a peak incidence in the sixth decade [15–16]. A considerable amount of patients (18%) had more than one HNBCC removed during the study period; in fact, as reported in a meta-analytic study, after the diagnosis of a first case of cBCC, the incidence of subsequent cases in the same patients increases by 10-fold compared to that of the general population [17].

As reported by most of the previous series [9, 12, 15–16, 18], also in our study the nasal unit (35%) was found to be the main site affected by the BCC, followed by the ear and the preauricular area (29%); the least frequent localization was instead the neck (2%). The high involvement of the nose as a subsite within the head and neck can be attributed to its anatomical structure, which is the most protruding part of the face and therefore most exposed to UV radiation [12]. There was no side predilection; 41% of BCCs were right-sided and 40% were left-sided. This result was different from our expectations, since in Italy the driver’s seat is on the left, therefore the left side of the face is subject to greater exposure to sunlight through the windows while driving. A prevalence of left-sided skin cancers, especially in the head and neck and in males, was instead found in the study by Burtler et al. [19].

The current retrospective audit showed clean skin margins in 251 (67%), positive in 80 (21%) and close in 46 (12%). These results are consistent with those reported in the literature for head and neck BCCs [9, 20], although some studies show lower rates of inadequate margins [7, 21] and others higher rates [13, 22]. In general, incomplete excision rates of BCCs are notoriously the highest in the head and neck area, due to the presence of noble anatomical structures of aesthetic and functional relevance that make surgical treatment challenging, with a tendency to use narrower margins [9, 23]. The more common involvement of the lateral margins, for both positive (65%) and closed margins (65%), was another finding coherent with the previous literature [6–7, 9, 14, 21]. The nasal site, as reported by several authors [6–7, 23], was also found to be the most frequent site of incomplete excision both for positive (49%) and for close margins (44%). The reasons for the common incomplete excision of nasal BCCs are due to the scarcity of available skin and excision usually limited to the suprachondral and perichondral layers; to reduce the risk of deformity, functional disability or the need for challenging reconstruction [9]. In comparison, other studies found a higher prevalence of positive margins in the forehead [9, 21] and ear [23].

In most patients in this cohort, the surgical wound was managed with direct closure (65%). This, as reported by other authors, is a consequence of the small size of the lesions at the time of excision (mean size 9.3 mm) and early diagnosis. In fact, the higher incidence of BCC in photoexposed areas, such as the head and neck, facilitates the early identification of smaller lesions, which do not require wider excisions and reconstructions [5, 12].

The treatment of the incompletely removed BCCs remains a controversial issue [5–6, 20, 24]. Our management policy for the 80 positive margins was mostly conservative with careful clinical observation, implementing re-excision only in 3 (4%) cases, all histologically free of residual BCC. This approach, in accordance with that of other authors such as Kumar et al. [5], was justified by the prevalence of elderly and frail patients with multiple comorbidities in our cohort. Furthermore, a histological report of positive margins does not equal with the presence of residual tumour or persistence of the disease [6]. After BCC excision surgical margins are often subjected to reworking such as cauterization to control haemostasis; moreover, during healing the scarring process and possible immune phenomena can lead to tumour regression phenomena [5, 14, 24]. Distinctly different, other authors have reported higher rates of re-excision with residual tumour rates ranging from 36 to 59% [6, 21].

Reviewing the recurrence rates of other series focusing on head and neck BCCs, ranging from 0 to 15% [4, 12, 16, 18, 20], the rate of our study cohort (5%) was found to be in line with the literature. Chow et al. found a mean time to recurrence of 36.6 months [20], which was higher than ours of 23.7 months; on the other hand, most of recurrences (54%) occurred in the first 2 years, as also reported in the study by Miszczyk et al. [24]. The median follow-up time of 35 months was also consistent with that of other studies [4, 15, 24] and sufficient to analyse recurrences.

Chi-square analysis of margin status showed no statistically significant association with tumour side (*p* > 0.05) and excision of multiple lesions in the same surgical session (> 0.05). In contrast, in the study by Masud et al., removal of the BCC via multiple lesion excision procedure rather than single lesion excision was related with a higher rate of incomplete excision [25]. Furthermore, no statistically significant associations were found between compromised margins and surgical experience (*p* > 0.05) of the seven head and neck consultants involved in the study. This finding was already reported by other series in the literature [5–6, 9], but differed from the results of Hansen et al. [23]. In this latest study, structurally different from ours as it compared the activity of 15 dermatology clinics in Australia and involved 57 physicians with different training backgrounds, the authors found substantial variation in frequency of incomplete excision of BCCs between clinics and surgeons. A statistically significant association was found between clean margins and direct wound closure (*p* < 0.05; adjusted residual 3.3) and between local flaps and positive-closed margins (*p* < 0.05; adjusted residual 2.1 and 2.0, respectively). These links can be explained by the fact that direct closure is usually applied for localized lesions in areas where skin is available and larger excisions can be performed. A local flap is instead necessary in locations where the skin availability is poor and the risk of aesthetic or functional damage is higher [5]. Conversely, Girardi et al. and Dalal et al. found no association between completeness of BCCs excision and type of reconstruction [7, 9]. Nasal area was significantly associated with positive margins (*p* < 0.05; adjusted residual 2.9), whereas clean margin status with neck site (*p* < 0.05; adjusted residual 2.1). These results were interesting because although several series [6, 9, 21, 23] reported a higher frequency of positive or clean margins at some specific head and neck sites, among those that performed Chi square analysis [7, 13], static insignificance was mostly found (*p* > 0.05). Besides, the depth of invasion below the dermis (*p* < 0.05; adjusted residual 2.5) was significantly correlated with positive margins, a risk factor for compromised margins of head and neck BCCs already highlighted in the study by Girardi et al. [7].

Even if the data of our cohort suggested a major trend toward a correlation between recurrence and positive margins (adjusted residual 2.1), no statistical significance emerged between margin status and recurrences (*p* > 0.05). Bourlidou et al. instead found that incomplete excision of the lesion predisposes to a twice higher risk of recurrence (RR = 2.5; 95% CI: 1.49–4.21; *p* < 0.001) in a study that included 531 basal cell carcinomas of the middle third of the face [16]. Recurrence was not found to be correlated with lateral, deep, or both location of the positive margin (*p* > 0.05). In contrast, Miszczyk et al. in a series of 156 incompletely excised head and neck BCCs observed a higher risk of recurrence in lateral margin involvement (RR = 1.24; 95% CI: 0.86–1.82; *p* = 0.22) [24]. Although depth of invasion was correlated with positive margins, this connection was not observed for recurrence (*p* > 0.05). A similar result was reported in the study by Bourlidou et al., in which depth of invasion was identified as a risk factor for recurrences for squamous cell carcinoma of the head and neck, but not for BCC [16]. The analysis highlighted a slight tendency towards a correlation between recurrence and nasal location (adjusted residual 2.2), although no statistical significance was found (*p* > 0.05). The same occurred in other reports in which the nose was the most frequent site of recurrence, without however finding a statistically significant association between primary tumour site and recurrence [15, 20]. Conversely, Miszczyk et al. reported a higher relative risk of recurrence in the scalp location (RR: 2.27; 95% CI: 1.14–2.27; *p* = 0.007) [24]. No correlations were also found between the method of wound closure and recurrence (*p* > 0.05); unlike Miszczyk et al. who observed a relative risk of 1.42 after closure with a split-thickness skin graft (RR: 1.42; *p* = 0.063; 95% CI: 0.94–1.95). However, the authors correctly pointed out that thickness skin grafts facilitate the diagnosis of recurrence, rather than directly influencing recurrence [24].

Finally, contrary to Girardi et al. who showed an association between tumour size and inadequate margins in head and neck BCCs [7], logistic regression analysis of our data did not find a correlation of tumour diameter with lateral, deep, or both margin result (*p* > 0.05), or with close, positive, or clean outcome (*p* > 0.05).

The limitations of this study are mainly related to its retrospective nature, which does not allow a precise selection of patients on predetermined factors and limits the more complex statistical or comparative analyses [26]. The monocentricity of the study and the relatively low number of patients represent another weak point. In addition, histological limitations were found for the presence of 21% of histological records indicating BCC with “indeterminate” histology. For these lesions, assessment of the reports revealed that only the dimensions and margin status were recorded. The study would have benefited from a comprehensive database with precise subtyping of BCCs. This lack did not allow investigating the influence of histological subtype on completeness of excision and risk of recurrence. Despite these limits, this study was conducted after a careful review of the literature and selecting all the variables analysed among the previous reports. This consented us to implement an investigation with satisfying statistical analysis and adequate patient follow-up time on a controversial multidisciplinary issue. It could guide prospective multicentre studies on larger populations to accurately outline the optimal treatment management for head and neck BCC.

## Conclusion

The status of surgical margins is affected by location, depth of invasion and method of reconstruction of head and neck BCC. These data are important both to guide the head and neck surgeon in the treatment of these skin cancers and for counselling the patient. Close clinical observation appears to be an adequate management choice in patients undergoing incomplete removal of head and neck BCCs, given of the low rates of recurrence. This is especially true in elderly and frail populations such as ours, where 75% of patients were over 70 years of age. Follow-up should be closer in the first 2 years, given our observation that 54% of recurrence occurred in this time frame. However, a 5-year follow-up is recommended to avoid missing any long-term recurrence.

## Electronic supplementary material

Below is the link to the electronic supplementary material.

Supplementary Material 1

## References

[1] Castanheira A, Boaventura P, Pais Clemente M, Soares P, Mota A, Lopes JM (2020) Head and neck cutaneous basal cell carcinoma: what should the otorhinolaryngology head and neck surgeon care about? Acta Otorhinolaryngol Ital 40(1):5–18. 10.14639/0392-100X-2245 31388193 10.14639/0392-100X-2245 PMC7147542

[2] Strom TJ, Caudell JJ, Harrison LB (2016) Management of BCC and SCC of the Head and Neck. Cancer Control 23(3):220–227. 10.1177/107327481602300305 27556662 10.1177/107327481602300305

[3] Szewczyk M, Pazdrowski J, Golusiński P, Dańczak-Pazdrowska A, Luczewski L, Marszałek S et al (2016) Basal cell carcinoma in farmers: an occupation group at high risk. Int Arch Occup Environ Health 89(3):497–501. 10.1007/s00420-015-1088-0 26464316 10.1007/s00420-015-1088-0 PMC4786594

[4] Lalloo MT, Sood S (2000) Head and neck basal cell carcinoma: treatment using a 2-mm clinical excision margin. Clin Otolaryngol Allied Sci 25(5):370–373. 10.1046/j.1365-2273.2000.00376.x 11012649 10.1046/j.1365-2273.2000.00376.x

[5] Kumar P, Orton CI, McWilliam LJ, Watson S (2000) Incidence of incomplete excision in surgically treated basal cell carcinoma: a retrospective clinical audit. Br J Plast Surg 53(7):563–566. 10.1054/bjps.2000.3394 11000071 10.1054/bjps.2000.3394

[6] Dieu T, Macleod AM (2002) Incomplete excision of basal cell carcinomas: a retrospective audit. ANZ J Surg 72(3):219–221. 10.1046/j.1445-2197.2002.02351.x 12071456 10.1046/j.1445-2197.2002.02351.x

[7] Girardi FM, Wagner VP, Martins MD, Abentroth AL, Hauth LA (2021) Factors associated with incomplete surgical margins in basal cell carcinoma of the head and neck. Braz J Otorhinolaryngol 87(6):695–701. 10.1016/j.bjorl.2020.02.007 32327363 10.1016/j.bjorl.2020.02.007 PMC9422635

[8] Lv R, Sun Q (2017) A Network Meta-Analysis of Non-melanoma skin Cancer (NMSC) treatments: Efficacy and Safety Assessment. J Cell Biochem 118(11):3686–3695. 10.1002/jcb.26015 28370183 10.1002/jcb.26015

[9] Dalal AJ, Ingham J, Collard B, Merrick G (2018) Review of outcomes of 500 consecutive cases of non-melanoma skin cancer of the head and neck managed in an oral and maxillofacial surgical unit in a District General Hospital. Br J Oral Maxillofac Surg 56(9):805–809. 10.1016/j.bjoms.2018.08.015 30219606 10.1016/j.bjoms.2018.08.015

[10] Slater D, Barrett P (2019) Dataset for histological reporting of primary cuta-neous basal cell carcinoma. In: The royal college of pathologist in the session: standards and datasets for reporting cancers. London. https://www.rcpath.org/static/53688094-791e-4aaa-82cec42c3cb65e35/Dataset-for-histopathological-reporting-of-primary-cutaneous-basal-cell-carcinoma.pdf. Accessed 26 November 2024

[11] Peris K, Fargnoli MC, Kaufmann R, Arenberger P, Bastholt L, Seguin NB et al (2023) European consensus-based interdisciplinary guideline for diagnosis and treatment of basal cell carcinoma-update 2023. Eur J Cancer 192:113254. 10.1016/j.ejca.2023.113254 37604067 10.1016/j.ejca.2023.113254

[12] Bertozzi N, Simonacci F, Greco MP, Grignaffini E, Raposio E (2019) Single center evidence for the treatment of basal cell carcinoma of the head and neck. Acta Biomed 90(1):77–82. 10.23750/abm.v90i1.6395 30889158 10.23750/abm.v90i1.6395 PMC6502149

[13] Janjua OS, Sana Qureshi SM (2012) Basal cell carcinoma of the head and neck region: an analysis of 171 cases. J Skin Cancer 2012:943472. 10.1155/2012/943472 23316370 10.1155/2012/943472 PMC3536434

[14] Herzum A, Burlando M, Tavilla PP, Micalizzi C, Molle MF, Cozzani E, Parodi A (2022) Dermatoscopically narrowed surgical margins for head and neck basal cell carcinoma: a retrospective case-control study. J Dtsch Dermatol Ges 20(6):807–816. 10.1111/ddg.14757 35581699 10.1111/ddg.14757 PMC9321004

[15] Demirseren DD, Ceran C, Aksam B, Demirseren ME, Metin A (2014) Basal cell carcinoma of the head and neck region: a retrospective analysis of completely excised 331 cases. J Skin Cancer 2014:858636. 10.1155/2014/858636 24864212 10.1155/2014/858636 PMC4016886

[16] Bourlidou E, Vahtsevanos K, Kyrgidis A, Tilaveridis I, Patsatsi A, Andreadis D et al (2019) Risk factors for local recurrence of basal cell carcinoma and cutaneous squamous cell carcinoma of the middle third of the face: a 15-year retrospective analysis based on a single centre. Eur J Dermatol 29(5):490–499. 10.1684/ejd.2019.3643 31789273 10.1684/ejd.2019.3643

[17] Marcil I, Stern RS (2000) Risk of developing a subsequent nonmelanoma skin cancer in patients with a history of nonmelanoma skin cancer: a critical review of the literature and meta-analysis. Arch Dermatol 136:1524–1530. 10.1001/archderm.136.12.1524 11115165 10.1001/archderm.136.12.1524

[18] Tourli I, Langner D, Haroske G, Tchernev G, Lotti T, Wollina U (2016) Basal cell carcinoma of the head-and-neck region: a single center analysis of 1,750 tumors. Georgian Med News.;(250):33–39. PMID: 26870972 26870972

[19] Butler ST, Fosko SW (2010) Increased prevalence of left-sided skin cancers. J Am Acad Dermatol 63(6):1006–1010. 10.1016/j.jaad.2009.11.032 20226568 10.1016/j.jaad.2009.11.032

[20] Chow VL, Chan JY, Chan RC, Chung JH, Wei WI Basal cell carcinoma of the head and neck region in ethnic Chinese. Int J Surg Oncol. 2011:2011:890908. 10.1155/2011/890908 10.1155/2011/890908 PMC3265281 22611492

[21] Patel SS, Cliff SH, Ward Booth P (2013) Incomplete removal of basal cell carcinoma: what is the value of further surgery? Oral Maxillofac Surg 17(2):115–118. 10.1007/s10006-012-0348-3 22868984 10.1007/s10006-012-0348-3 PMC3661037

[22] Wollina U, Bennewitz A, Langner D (2014) Basal cell carcinoma of the outer nose: overview on surgical techniques and analysis of 312 patients. J Cutan Aesthet Surg 7(3):143–150. 10.4103/0974-2077.146660 25538434 10.4103/0974-2077.146660 PMC4271293

[23] Hansen C, Wilkinson D, Hansen M, Soyer HP (2009) Factors contributing to incomplete excision of nonmelanoma skin cancer by Australian general practitioners. Arch Dermatol 145(11):1253–1260. 10.1001/archdermatol.2009.270 19917954 10.1001/archdermatol.2009.270

[24] Miszczyk J, Charytonowicz M, Dębski T, Noszczyk B (2017) Incomplete excision of basal cell carcinoma (BCC) in the head and neck region: to wait, or not to wait? Postepy Dermatol Alergol 34(6):607–611. 10.5114/ada.2017.72467 29422827 10.5114/ada.2017.72467 PMC5799764

[25] Masud D, Moustaki M, Staruch R, Dheansa B (2016) Basal cell carcinomata: risk factors for incomplete excision and results of re-excision. J Plast Reconstr Aesthet Surg 69(5):652–656. 10.1016/j.bjps.2015.12.024 26948998 10.1016/j.bjps.2015.12.024

[26] Iocca O, Copelli C, Rubattino S, Sedran L, Di Maio P, Arduino PG et al (2023) Oral cavity carcinoma in patients with and without a history of lichen planus: a comparative analysis. Head Neck 45(6):1367–1375. 10.1002/hed.27350 37002194 10.1002/hed.27350

