# Supplementary material for: Head and neck cutaneous basal cell carcinoma: a retrospective analysis of tumour features, surgical margins and recurrences
Source: Eur Arch Otorhinolaryngol. 2025 Feb 20;282(6):3183–91. doi: 10.1007/s00405-025-09216-z (PMC12122600; doi:10.1007/s00405-025-09216-z)
Supplement: Supplementary file 1 — Supplementary Material 1 [file 405_2025_9216_MOESM1_ESM.pdf]

Sistema Sanitario Regione Liguria

Otorinolaringoiatria - Sanremo

Dipartimento Area Chirurgica

Direttore: Dott. Marco Giudice

To whom it may concern:

This declaration is to certify that at this Institution there is a policy to waive the necessity of asking for Ethical Committee approval for studies with a retrospective design.

All the information are available on the ethical committee website (in Italian): https://www.ospedalesanmartino.it/it/ricerca-scientifica/sperimentazioni-cliniche/informazioni-cro-promotori/documentazione-richiesta-per-la-valutazione.html

Best regards,

Dott. Marco Giudice

Director – Surgical Department and Otorhinolaryngology Department

Sanremo Civic Hospital

Italy
